# Low-Velocity Gunshot Femur Fractures Do Not Require Surgical Debridement

**DOI:** 10.3390/jcm15114278

**Published:** 2026-06-01

**Authors:** Jessica Bear, Mary-Kate Erdman, Lane Shepherd, Charalampos G. Zalavras

**Affiliations:** Department of Orthopaedic Surgery, Los Angeles General Medical Center, University of Southern California, Los Angeles, CA 90033, USA

**Keywords:** femur fractures, femoral fractures, gunshot, ballistic, low velocity, debridement, infection, union

## Abstract

**Background/Objectives:** Management of low-velocity gunshot fractures is controversial, and the need for surgical debridement is being debated. This study aimed to compare the infection and union rate of low-velocity gunshot femur fractures treated without formal debridement to those of closed femur fractures due to blunt trauma. **Methods:** This is a retrospective comparative study of patients with low-velocity gunshot femur fractures to age- and gender-matched controls with closed femur fractures due to blunt trauma. All patients were treated with intramedullary nailing without exposure and debridement of the fracture site. Outcome variables included fracture-related infection, superficial infection, and union. Statistical significance was set at *p* < 0.05. **Results:** The gunshot group consisted of 26 patients (96% male, median age 23 years) and the control group of 52 patients (96% male, median age 23 years. The gunshot group had a significantly lower proportion of additional musculoskeletal injuries compared to the control group (15% vs. 48%, *p* = 0.006) and a higher proportion of smoking (70% versus 27%, *p* = 0.001). There were no fracture-related infections in the gunshot group, whereas there was one (2%) in the control group, which was not statistically significant (*p* = 1.00). There were no superficial infections in either group. All fractures were united in both groups. **Conclusions:** Low-velocity gunshot femur fractures treated with antibiotics but without debridement have similar infection and union rates as closed femur fractures due to blunt trauma. Low-velocity gunshot femur fractures behave as closed fractures, and formal surgical debridement is not indicated.

## 1. Introduction

Civilian gunshot injuries are a very important health care issue worldwide, especially in the United States, with more than 48,000 firearm-related deaths in 2022 according to the Center for Disease Control mortality data [1]. Firearm-related injury rates are difficult to measure due to challenges with collecting national injury data, but studies suggest that there are at least twice as many nonfatal firearm injuries as fatal ones [2]. The majority of gunshot wounds (GSWs) involve the extremities, with fractures commonly present [3,4].

Gunshot wounds are traditionally classified by a velocity threshold of 2000 feet per second as low-velocity (<2000 ft/s) and high-velocity (>2000 ft/s) wounds. In low-velocity wounds, which represent most gunshot wounds encountered in civilian trauma settings, tissue destruction is largely confined to structures physically contacted by the projectile, and soft tissue disruption is generally limited. On the other hand, high-velocity wounds, which are usually encountered in combat trauma settings, are characterized by extensive soft tissue injury well beyond the bullet path [5].

The core debate centers on whether low-velocity civilian gunshot fractures require formal operative debridement, as traditionally applied to open fractures, or can be safely managed with superficial wound care alone. Proponents of formal surgical debridement, even for low-energy civilian gunshot fractures, argue that bullets are not sterilized on discharge [6], bullets can transport bacteria from the skin flora and clothing directly into the wound [7], and retained bullet fragments have been associated with a significantly increased risk of FRI [8]. Furthermore, fracture-related infection (FRI) rates after gunshot injuries have ranged from 3.6% to 22% in some studies [9].

On the other hand, accumulating civilian data has challenged the principle of formal operative debridement for low-velocity GSWs, suggesting that these injuries behave more like closed fractures rather than open fractures. Very low infection rates have been reported in the absence of formal surgical debridement [10,11,12,13,14,15], even without the administration of antibiotics [13,16,17].

Without widely accepted indications for surgical debridement and protocols on antibiotic administration, the management of civilian GSW fractures remains controversial, despite their high number [18,19,20,21,22]. The femur is the long bone most commonly involved in gunshot fractures [3], but, to our knowledge, no study has directly compared the outcome of ballistic fractures to that of closed fractures of the femur due to blunt trauma in matched cohorts.

The aim of the current study was to compare patients with low-velocity gunshot femur fractures treated with intramedullary nailing (IMN) but without formal debridement to a matched cohort of patients with closed femur fractures identically treated with IMN to investigate the effect of the mechanism of injury and absence of formal debridement on the infection and union rates. Our null hypothesis was that there would be no difference in the infection and union rates between the two groups. Clarifying these issues would help optimize patient management, which would be of considerable benefit to patients, health-care providers, and society in general, given the enormous socioeconomic burden of these injuries [23].

## 2. Patients and Methods

### 2.1. Study Design

Institutional review board approval was obtained for this retrospective study. We searched our institution’s trauma database for patients with femoral diaphyseal fractures (OTA/AO 32 according to the OTA/AO classification [24]) sustained from a low-velocity gunshot injury, who were treated at our level I trauma center, with closed reamed IMN without formal surgical debridement of the fracture site between 21 July 2007 and 11 November 2016.

Our exclusion criteria were patients with incomplete medical records, patients with open physes, patients who underwent ipsilateral vascular repair or reconstruction even if the fracture site was not surgically exposed, patients who developed compartment syndrome, patients who underwent formal surgical debridement of the fracture site, and patients with a follow-up less than 3 months postoperatively, unless an infection was diagnosed at an earlier time.

After identification of the study group, an age- and gender-matched control group was randomly selected from closed femur fractures treated by closed reamed IMN at our institution during the same time period, using all the exclusion criteria of the study group, with the addition of pathologic and bisphosphonate-related fractures. The exclusion of fractures complicated by vascular injury or compartment syndrome from both groups and the fact that our control group consisted of closed fractures (without inclusion of any open fractures due to blunt trauma) aimed to avoid the confounding effect of these factors and create two homogenous groups with the mechanism of injury (GSW versus blunt trauma) as the main variable that differed between them. Controls were matched to cases using a 2:1 ratio.

### 2.2. Patient Management

All fractures in the study were treated by or under the direct supervision of an attending of our orthopedic trauma service. The decision not to perform formal surgical debridement of gunshot fractures and the choice of antegrade versus retrograde IMN nail insertion were based on surgeon preference. All fractures in the study were reduced in a closed manner without exposure, visualization, or debridement of the fracture site.

All patients with gunshot femur fractures were treated with antibiotics for 24 to 72 h based on the surgeon’s preference. All patients with closed fractures received standard perioperative antibiotic prophylaxis for 24 h.

Postoperatively, patients were allowed free hip and knee range of motion, unless a concomitant injury precluded this. Weight-bearing status varied depending upon associated injuries, fracture characteristics, and surgeon preference.

### 2.3. Outcome Variables

The diagnosis of fracture-related infection was based on one or more of the following criteria of the consensus definition as proposed by Metsemakers et al. [25]: sinus or wound breakdown with communication to the bone or the implant, purulent drainage from the wound or presence of pus during surgery, phenotypically indistinguishable pathogens identified by culture from at least two separate intraoperative deep specimens, and microorganisms in deep tissue identified by histopathological examination using specific staining techniques.

An infection involving only the skin and subcutaneous tissues of the surgical or gunshot wounds, noted to be superficial to the fascia, that completely resolved with oral antibiotics and local wound care was considered a superficial one.

Fracture healing was evaluated in postoperative radiographs by three independent reviewers (two orthopedic traumatologists and one senior resident). Fractures were considered healed when there was evidence of bridging callus involving at least three of four cortices on two radiographic projections, agreed upon by at least two of the three reviewers. Fractures were also considered healed in patients with bridging callus involving at least two of four cortices, who had further clinical but not radiographic follow-up and were asymptomatic at more than one year postoperatively.

### 2.4. Statistical Analysis

Continuous data are presented as median [interquartile range (IQR)]. The Mann–Whitney U test was used for the comparison of continuous variables between the two groups. The Fisher’s exact test was used for comparison of categorical variables between the two groups. Statistical significance was set at *p* < 0.05. All *p* values were two-tailed.

## 3. Results

We identified 78 patients with femoral diaphyseal fractures from a low-velocity gunshot injury, who were treated by reamed IMN. We subsequently excluded patients with incomplete medical records (n = 6), patients with open physes (n = 1), patients who underwent ipsilateral vascular repair or reconstruction (n = 4), patients who underwent debridement of the fracture site (n = 28), and patients who failed to follow up (n = 13). Therefore, our gunshot study group consisted of 26 patients.

Twenty-five (96%) of the 26 patients were male and one (4%) was female. The median age was 23 years [IQR, 19 to 29]. Nineteen patients (73%) had isolated femoral fractures, and seven had additional injuries, including concomitant orthopedic injuries in four patients and abdominal injuries in three. Seventeen patients (65%) were treated with an antegrade femoral nail, while nine patients were treated with a retrograde femoral nail. The median follow-up time was 5.2 months [IQR, 4.0 to 9.9].

Our control group consisted of 52 patients. There were 50 (96%) male patients and two (4%) female patients with a median age of 23 years [IQR, 20 to 30]. The median follow-up time was 6.1 months [IQR, 4.1 to 11.4]. The characteristics of the control group and comparison with the GSW group are presented in Table 1. The gunshot group had a significantly lower rate of additional musculoskeletal injuries compared to the control group (15% versus 48%, *p* = 0.006). Smoking was significantly more prevalent in the gunshot group (70% versus 27%, *p* = 0.001).

There were no fracture-related infections in the gunshot group, whereas there was one fracture-related infection (2%, 1 of 52) in the control group. The difference was not statistically significant (*p* = 1.00). This control patient developed a fracture-related infection in a healed fracture, presented with drainage from the distal locking screws and underwent implant removal and debridement 8 years after the index procedure.

There were no superficial infections in either group. No patient treated with retrograde IMN developed septic arthritis of the knee.

A post hoc power analysis showed that our study was underpowered to detect a significant difference in infection rates between the two groups. Assuming an infection rate of 2.5% in gunshot femur fractures [13,26] and 1% in closed femur fractures [27,28], a sample size of 2535 patients (845 in the GSW group and 1690 in the control group) would have been needed to achieve 80% power (beta 0.80) with alpha 0.05.

All fractures were united in both groups without requiring subsequent surgery to achieve union, and there was no difference in the union rates (100% in each group, *p* = 1.00).

## 4. Discussion

High-velocity GSWs, as seen in combat injuries, routinely cause extensive soft tissue damage and are associated with high infection rates; therefore, it is generally accepted that fractures caused by such injuries should undergo formal surgical debridement prior to or at the time of fracture stabilization [29,30].

Low-velocity GSWs, as seen in civilian practice, create a much smaller amount of soft tissue injury. Nevertheless, bullets are not autosterilized upon firing, and they introduce skin flora into the wound, invariably resulting in contamination of the wound track [6,31]. This creates a rationale for antibiotics and formal surgical debridement of these injuries. However, very low infection rates, approximately 0% to 3%, have been reported in stable low-velocity gunshot fractures treated without formal surgical debridement [10,11,12,13,14,15], even without the administration of antibiotics [13,16,17].

Similarly, low infection rates have been reported in unstable low-velocity femur fractures surgically treated with fixation but without exposure of the zone of injury and without formal surgical debridement of the fracture site [26,32,33,34,35,36,37]. Nowotarski reported a 2.6% deep infection rate in patients who underwent IMN without formal debridement for femur fractures caused by low-velocity gunshots [26]. Hollmann and Horowitz reported a 3.8% superficial infection rate and no deep infections [32], whereas other authors reported no infections at all in the patients treated with IMN without formal debridement [33,37].

Authors of an older literature review [21] were not able to recommend for or against extensive debridement for low-velocity gunshot fractures requiring surgical fixation, and subsequent surveys have shown that optimal management of these injuries is not universally agreed upon [19,20]. Nguyen et al. reported that 68% of surveyed OTA members would treat an unstable tibia shaft fracture—with no additional skin opening other than the GSW—as an open fracture with surgical debridement and fixation of the fracture, whereas 28% would only fix the fracture without surgical debridement [20]. As the authors stated, this variability in practice demonstrates insufficient understanding of the characteristics of fractures due to GSWs and poses the question of whether unstable gunshot fractures behave like open fractures or closed fractures [20]. A more recent review of civilian low-velocity gunshot extremity injuries reported that management of these injuries is still debated [22].

Our study demonstrates that low-velocity gunshot femur fractures treated without formal surgical debridement have very low infection rates and very high union rates that are similar to those of closed femur fractures due to blunt trauma. We want to point out that the rates of 0% and 2% do not necessarily confirm equivalence. However, both infection rates were very low, and the 2% infection rate was in the blunt trauma group (for which the management is standardized and non-controversial), whereas in the GSW group treated without debridement (the group around which the controversy revolves), the infection rate was 0%, thereby adding support to not performing formal surgical debridement in this group.

Our findings support the hypothesis that low-velocity ballistic fractures behave like closed fractures, and as such, do not require formal surgical debridement.

The very low infection rates in low-velocity gunshot fractures, despite the contamination present and the omission of debridement, may be attributed to the administration of antibiotics and to the protective effect of host defense mechanisms. The systematic review by Papasoulis et al. [13] reported significantly lower infection rates in gunshot fractures treated nonoperatively when antibiotics were given compared to no antibiotics (2.4% vs. 6.7%). When only higher-quality studies were assessed, a similar reduction in infection rates was observed (1.7% vs. 5.1%), but it was not significant with the numbers available [13]. Nevertheless, the majority of patients with low-velocity gunshot fractures treated without antibiotics do not develop infections [13,17], which underscores the importance of the viable surrounding soft tissue envelope delivering blood supply to the fracture site.

In the setting of low-velocity gunshot injuries, it may be argued that performing an extensive formal debridement with removal of the bullet(s) may still be beneficial by further decreasing the already very low infection rate. However, this may not be the case; first, in low-velocity gunshot fractures, the surrounding soft tissues remain viable, and second, extensive bullet fragmentation is often present, making removal of all foreign material impractical and potentially harmful if an exhaustive search for each bullet fragment is undertaken. Therefore, formal surgical debridement may incur a biological cost without an associated benefit. Interestingly, a recent study reported higher infection rates in low-velocity gunshot fractures of the tibia treated with formal debridement compared to those treated without [38].

Two recent studies compared low-velocity gunshot femur fractures to blunt trauma femur fractures [39,40] with considerable variability in the reported rates of FRI. Patch et al. reported that the ballistic femur fracture group had a higher rate of FRI compared to the blunt closed femur fracture group (6.0% vs. 4.0%), but this did not reach statistical significance [39]. On the other hand, Donahue et al. reported an FRI rate of 0.7% in the ballistic femur fracture group vs. 1.5% in the blunt femur fracture group [40]. It should be noted that in the study by Patch et al., 13% of the GSW fractures were complicated by compartment syndrome and none by arterial injury, whereas in the study by Donahue et al., 6% of GSW fractures were complicated by compartment syndrome and 9% by arterial injury [39,40]. The variability in the reported rates of FRI may be explained by the underlying differences in associated complications and their confounding effect on the development of infection.

A unique strength of our study is the application of strict exclusion criteria for both the gunshot and the blunt fracture groups. The exclusion of fractures complicated by vascular injury or compartment syndrome from both groups and the exclusion of open fractures from the blunt trauma group avoided the confounding effect of these factors and created two homogenous groups, which were treated in an identical fashion with closed IMN without exposure or debridement of the fracture site. Furthermore, the two groups were matched by age and gender. Therefore, the mechanism of injury (GSW versus blunt trauma) was the main variable that differed between groups, and the effect of any confounding variables was minimized. This is the first study, to our knowledge, to directly compare infection rates between low-velocity ballistic fractures and closed fractures of the femur in such a way.

There are important limitations in this retrospective study. First, the median follow-up of patients was limited to 5.2 months in the gunshot group and 6.1 months in the control group, and the additional FRIs may have developed at a later time and not been captured by our study. It should be noted that our hospital serves an underprivileged and migratory population with socioeconomic barriers to obtaining routine follow-up care, such as incarceration, lack of insurance, and undocumented immigration status. However, all patients included in the study had a minimum 3-month follow-up, and the follow-up time did not differ between the two groups.

Second, there was no uniform antibiotic treatment protocol in our gunshot femur fracture group during the 11-year period of the study. However, all patients with such fractures were consistently treated with antibiotics for 24 to 72 h. A systematic review documented that the exact antibiotic duration and regimen did not alter the infection rate in gunshot fractures [9].

Third, our findings may not be applicable to gunshot femur fractures complicated by vascular injury or compartment syndrome, or to gunshot fractures at other anatomic locations with a thin, soft tissue envelope, such as the distal tibia. Our results may also not be generalizable to older patients and women. However, the vast majority of gunshot injuries are consistently sustained by young men in the literature, which makes the findings of our study applicable to most patients seen in clinical practice.

Fourth, there was an imbalance in smoking and musculoskeletal injuries between the two groups, and the bullet retention was not recorded, which may have confounded the results.

Finally, inter-observer reliability for the assessment of union was not assessed, and the union time was not calculated because not all patients had radiographs at similar and comparable time points.

In conclusion, low-velocity gunshot femur fractures treated with IMN and antibiotics but without debridement of the fracture site have very low infection rates and very high union rates, similar to those of closed blunt-trauma fractures in age- and gender-matched controls. It appears that low-velocity gunshot fractures to the femur behave like closed femur fractures, and as such, do not require formal surgical debridement.

## Figures and Tables

**Table 1:** Patient data for the GSW and the control group.

	GSW Group (n = 26)	Control Group (n = 52)	Comparison Between Groups (*p* Value)
Male gender	25 (96%)	50 (96%)	1.00
Age (years)	23 [19–29]	23 [20–30]	0.44
Additional injuries *	7 (27%)	28 (54%)	0.03
Other musculoskeletal injuries	4 (15%)	25 (48%)	0.006
Abdominal injuries	3 (12%)	5 (10%)	1.00
Chest injuries	1 (4%)	5 (10%)	0.66
Head/facial injuries	0 (0%)	4 (8%)	0.29
Sciatic nerve palsy	1 (4%)	0 (0%)	0.33
Diabetes mellitus	1 (4%)	2 (4%)	1.00
Smoking **	14 (70%)	14 (27%)	0.001
Antegrade IMN	17 (65%)	35 (67%)	1.00

Data are presented as no (%) for categorical variables and as median [IQR] for continuous variables. GSW: gunshot wound and IMN: intramedullary nailing. * some patients sustained more than one type of additional injury. ** smoking status had not been documented in six patients in the GSW group; 14 (70%) of the 20 patients with documented smoking status were smokers.

## Data Availability

The data presented in this article are not readily available because they are part of an ongoing study.
